# Ingested Type I Interferon—State of the Art as Treatment for Autoimmunity Part 2

**DOI:** 10.3390/ph3041108

**Published:** 2010-04-14

**Authors:** Staley A. Brod

**Affiliations:** Multiple Sclerosis Research Group, Department of Neurology, MSB 7.044, 6431 Fannin St., University of Texas Health Science Center at Houston, Houston, TX 77030, USA; E-Mail: staley.a.brod@uth.tmc.edu; Tel.: +001-713 500-7046; Fax: +1-713 500-7040

**Keywords:** autoimmunity, multiple sclerosis, Type I diabetes, Type I interferon, GALT, protein ingestion

## Abstract

We have proposed a unifying hypothesis of the etiopathogenesis of autoimmunity that defines autoimmunity as a type I interferon (IFN) immunodeficiency syndrome. We have examined toxicity and potential efficacy in two phase I (type 1 diabetes [T1D], multiple sclerosis [MS]) and phase II clinical trials in T1D and MS. In a phase I open label trial in T1D, ingested IFN-alpha preserved residual beta-cell function in recent onset patients. In a second phase I trial in MS, there was a significant decrease in peripheral blood mononuclear cell IL-2 and IFN-gamma production after ingesting IFN-alpha. In a phase II randomized, placebo-controlled, double-blind trial in MS, 10,000 IU ingested IFN-alpha significantly decreased gadolinium enhancements compared to the placebo group at month 5. TNF-alpha and IFN-gamma cytokine secretion in the 10,000 IU group at month 5 showed a significant decrease that corresponded with the effect of ingested IFN-alpha on decreasing gadolinium enhancements. In a phase II randomized, placebo-controlled, double-blind trial in T1D, patients in the 5,000 unit hrIFN-alpha treatment group maintained more beta-cell function one year after study enrollment compared to individuals in the placebo group. Ingested IFN-alpha was not toxic in these clinical trials. These studies suggest that ingested IFN-alpha may have a potential role in the treatment of autoimmunity.

## Introduction

We have proposed a unifying hypothesis of the etiopathogenesis of autoimmunity that defines autoimmunity as a type I IFN immunodeficiency syndrome [1]. Two major diseases, MS and T1D are thought to be autoimmune diseases characterized by T lymphocyte delayed type hypersensitivity (DTH) responses, differing in their target organs: the brain and beta islet cell, respectively. Because the pathogenic antigen is unknown in these auto-immune diseases [2], and assuming our hypothesis is correct, supplementation with type I IFN may be a therapeutic option. Ingested type I IFN may show therapeutic efficacy [3] and have significant advantages [4]. We have examined toxicity in two phase I (MS, type 1 diabetes) and potential efficacy in two phase II clinical trials in T1D and MS. 

MS is a chronic demyelinating disease of the CNS, which has been postulated to be a T cell mediated auto-immune disease [5]. MS is clinically associated with periods of disability (relapse) alternating with periods of recovery (remission) but often leading to progressive neurological disability [6]. MS has been associated with abnormalities of immuno-regulation [7]. MS stands out as the most common and intensely investigated demyelinating human disease. In aggregate, enormous medical resources are required to manage MS. The etiology and pathogenesis of MS are still far from unraveled. 

Interventions that reduce clinical activity in MS also significantly decrease enhancing lesions on brain MRI scans. Injectable (parenteral) IFN-betã1b (Bestaseron^®^) decreases relapses by 30% and decreases enhancing lesions by 80% in RRMS [8]. IFN-betã1a (Avonex^®^) by I.M. injection reduces progression by 37%, relapse rate by 18% reduction (ITT analysis), and Gd-enhancing lesions by 33% in relapsing remitting MS (RRMS) [9]. Intramuscular IFN-alpha a (Roferon^®^) treatment results in fewer new MRI lesions during the treatment period [10,11] and fewer clinical signs of disease activity in RRMS [11]. There is evidence that decreasing Gd-enhancements may have a positive effect on long term outcome in MS [12,13]. However, adverse events occur in up to 60% of patients receiving parenteral type I IFN, sometimes requiring discontinuation of treatment [9,10,14]. The use of parenterally administered type I IFN in early RRMS is limited by the generation of IL-6, a potential polyclonal B cell activator [15,16]. Furthermore, 40% of IFN-beta_1b_ treated patients generated neutralizing antibodies which are frequently found in those patients who appear to lose both clinical benefits and MRI-defined responses [14]. 

T1D is a chronic disorder that results from presumed autoimmune destruction of the insulin-producing pancreatic beta cell. In the United States, the prevalence of T1D by age 20 years is 0.26% and lifetime prevalence approaches 0.40%; thus 1.5 million Americans have T1D [17]. Histologic studies suggest that a significant reduction in the volume of beta cells is required to induce symptomatic T1D [17]. Intervention at clinical onset of disease is designed to prolong the period of residual beta cell function, recognized clinically as a “honeymoon” (a period in which the insulin need remains minimal and glycemic control improves, probably due to partial recovery of the insulin producing beta cell). However, when this period ends, the patient becomes completely insulin-deficient and dependent on exogenous insulin replacement. The international diabetes community agrees on the need to test potential preventive therapies for T1D in newly-diagnosed patients. Interventions prolonging the honeymoon period, indicative of the reversal of the disease, are considered positive [18]. Numerous interventions have attempted to spare residual insulin activity. In the Diabetes Control and Complications Trail (DCCT), experimental intense insulin therapy produced less decline in stimulated C-peptide values [19]. In patients with diabetes for more than five years, 11% (33 of 296) of adults, compared with 0 of 75 adolescents, retained substantial insulin secretory capacity [19]. Intensive, continuous insulin treatment during the first two weeks after the diagnosis of T1D mellitus may improve beta-cell function during the subsequent year [20]. Over the past 25 years, multiple clinical trials attempted to prevent progressive beta-cell destruction after the diagnosis of T1D using immunosuppressive or immunomodulatory agents such as cyclosporine [21,22], cyclosporin in combination with bromocriptine [23], azathioprine with or without glucocorticoids [18,24], nicotinamide with or without glucocorticoids [25,26] and parenteral IFN-alpha [27]. More recent intervention trials used monoclonal antibody based therapies such as rituximab (anti-CD-20) [28], daclizumab (anti-CD25) in combination with mycophenylate mofetil [29], anti-CD3 [30,31,32], and glutamate acid decarboxylase (GAD) [33]. While several studies are still ongoing and thus the results pending, trials of anti-CD3 antibodies and GAD have demonstrated delayed decline of endogenous insulin secretion. However, none of these intervention trials have yet been translated into common clinical practice for two reasons: (1) The beta-cell protective effect has been temporary and (2) some of the agents available were associated with unacceptable side effects, such as impairment of renal function in the case of calcineurin inhibitors. The natural history of T1D demonstrates that 90–97% of T1DM patients spontaneously end honeymoon period within one year of diagnosis. In light of the above, there is no effective treatment for T1D. 

In 1957 Isaacs and Lindenmann described a factor (interferon [15]) produced by virus infected cells with rapid antiviral activity [34]. Type 1 IFN is composed of two homologous (50%) proteins [35] IFN-alpha (leucocyte IFN) and IFN-beta (fibroblast IFN) with similar biological properties [36]. Acid stable natural alpha interferons contain 165–166 amino acids with about 80% sequence homology to each other [37,38]. IFN-alpha and IFN-beta are relatively similar in their actions and interact with the same cell receptor [39,40]. 

The administration of cytokines via the gut offers an exciting alternative to systemic application due to ease of dispensation in clinical use [41], patient convenience [42], ease of delivery, tolerance, and low cost along with a favorable therapeutic index [4,43]. Ingested IFN-alpha probably works by different mechanism than does parenterally administered type I IFN. Type I IFN are acid stable and most likely resist pre-prandial stomach acidity. When administered in the morning before eating, without digestive enzymes, type I IFN can survive passage to the small bowel. This is an important segment of the gut associated lymphoid tissue (GALT) consisting of lymphoid nodules termed Peyer's patches [44], a site where regulatory cells can be generated [45,46]. Fifty to two hundred high affinity type I IFN receptors are found on all lymphoid cells, including those of the GALT [47,48]. Mice given oral type I IFN show a systemic neutropenia. Circulating specific antibody to IFN blocks the neutropenic effects of parenteral IFN-alpha, but not the neutropenic effects of oral IFN [49]. Oral administration of IFN-alpha to mice [50], rabbits [51], dogs [52], monkeys [53], and humans [42] in doses exceeding one billion IU do not result in detectable levels of IFN-alpha in the blood. Up to 48 hours after 10^9^ IU IFN-beta was ingested by humans, neither serum IFN, beta_2_-microglobulin, neopterin, nor 2–5A synthetase were increased [42]. If ingested IFN-alpha is not absorbed, how does IFN-alpha transduce its signal?

## Experimental Section

In order to examine the possible mechanism of transduction of an IFN signal across the gut wall, we examined MxA message in lymphocytes after ingestion of IFN-alpha. MxA is a type 1 IFN-specific induced mRNA/protein, thus providing a marker indicating type I IFN/type I IFN receptor interaction (IFNAR) [54]. Induction of Mx mRNA is found in the absence of detectable serum IFN activity, demonstrating that MxA gene expression is a good marker for detecting minute quantities of biologically active type I IFN [55]. Ingested type 1 IFN must act through type I IFN receptors to transduce signals to immuno-modulatory cells [39]. We examined the relative levels of MxA mRNA signal using semi-quantitative RT-PCR on splenocytes from mice and PMNC from man after IFN-alpha ingestion. Both mice spleen cells (4 fold) and human PMNC demonstrated significant inducible levels of Mx mRNA after ingesting IFN-alpha. Murine whole splenocytes demonstrated up-regulation of MxA mRNA after IFN-alpha ingestion of 10 and 100 IU, clinically effective doses in EAE, but not after 1000 or 5000 IU, clinically ineffective doses in experimental autoimmune encephalomyelitis (EAE) [56]. Ingested IFN-alpha acts via established pathways of type 1 IFN signaling [57]. 

We have repeatedly and reproducibly shown that ingested IFN-alpha is a robust biological response modifier (BRM) in EAE [56,57,58,59,60]. We determined that ingested IFN-alpha was non-toxic and had biological effects in humans in a phase I study. Ingested hrIFN-alpha showed no toxicity in normal volunteers or patients with relapsing-remitting MS (RRMS) at doses ranging from 300 to 100,000 IU. In subjects with RRMS, a significant decrease in mitogen-induced peripheral blood mononuclear cells (PMNC) proliferation and serum soluble intercellular adhesion molecule-1 (sICAM-1), a surrogate measure for disease activity in MS, was found after ingesting six doses every other day of 10,000 and 30,000 IU IFN-alpha. Others have shown that IFN-alpha can establish a Th1-like cytokine bias in humans [61,62,63]. The RRMS subjects also showed decreased mitogen-induced IL-2 secretion after ingesting 10,000 IU IFN-alpha and decreased IFN-gamma, TGF-beta and IL-10 production after ingesting 30,000 IU IFN-alpha. The decreased secretion of IFN-gamma and IL-2, Th1-like cytokines, suggests that ingested IFN-alpha may inhibit predominantly pro-inflammatory Th1- like T helper cells in RRMS, a potential site of intervention at the level of effector T cells in MS. The above phase I study supported the oral use of human IFN-alpha as a biological response modifier in humans [64].

Subsequently, we investigated if ingested hrIFN-alpha was safe and if the treatment could reduce the number of gadolinium-enhanced lesions on serial cerebral MRI in patients with active RRMS. Serial MRI detects 5–10 times more disease activity in RRMS and secondary progressive MS patients than is clinically apparent and consequently is a sensitive tool with which to monitor disease activity [65,66,67,68,69,70]. Entry criteria included clinically definite RRMS and one or more gadolinium-enhanced lesions on a screening MRI. Eighty patients were screened, 33 found eligible and 30 patients were enrolled for treatment, 10 in each treatment arm. Eligible patients were randomized to treatment with placebo, 10,000 or 30,000 IU IFN-alpha2a ingested on alternate days for nine months. They were evaluated clinically and with monthly cerebral MRI. Sample size projections were based on the assumption of a parenteral “IFN-like effect”, a 90% reduction of enhancements evident within one month of the initiation of treatment in the active treatment groups sustained over the nine month study. This was not observed. However, data analysis showed a treatment effect in the 10,000 IU group. By direct monthly comparison of placebo and 10,000 IU group in treatment month 5, there were significantly fewer enhancements in the 10,000 IU group compared to the placebo group. The cumulative mean number of enhanced lesions showed a decrease in the 10,000 IU group compared to placebo starting at three months and continuing until six months. Analysis of recall antigen tetanus toxoid-stimulated PMNC TNF-alpha cytokine secretion in the 10,000 IU group showed a significant decrease compared to placebo that corresponded with the apparent effect of 10,000 IU ingested IFN-alpha2a on decreasing gadolinium enhancements. IFN-gamma cytokine secretion showed a clear downward trend in the low dose group compared to placebo at month 5. The combined data from the phase I and II trials do not suggest a Th1-cytokine bias after ingested IFN-alpha. Relapses and adverse events were not different among the treatment groups. Ingested IFN-alpha a did not induce systemic anti-IFN-alpha antibodies. These results suggest that doses lower than 10,000 IU may be necessary for clear efficacy because of tachyphylaxis or pharmacological tolerance at 10,000 IU [71]. Therefore, we examined MxA mRNA induction and TNF-alpha mRNA repression after 100, 300, 1,000, 3,000 and 10,000 IU doses of ingested IFN-alpha in 24 RRMS patients to determine the optimal dose(s) for future clinical trials in MS. Maximal TNF-alpha repression occurs at 100, 1,000 and 3,000 IU. ). These findings suggest that ingested interferon may have a hormetic dose response relationship (*i.e*., generally favorable biological responses to low exposures with the opposite effect at high doses). The biology underlying such biphasic dose-response relationships is poorly understood, but they appear to be common in immunology [72]. These data provide new optimal doses for additional clinical studies using ingested IFN-alpha in MS and show that lower doses have greater activity [73].

We previously determined that ingested murine IFN-alpha (mIFN-alpha) administered to non-obese diabetic (NOD) mice decreased islet inflammation and suppressed T1DM [74]. Ingestion of mIFN-alpha increased the mitogen-induced production of IL-4, IL-10 (Th2-like cytokines) and IFN-gamma secretion in spleen cells from treated mice. Adoptive transfer of unstimulated splenocytes secreting IL-4 and IL-10 from mIFN-alpha fed donors suppresses spontaneous T1DM in recipients. The protective effect of adoptively transferred unstimulated splenocytes demonstrates the presence of ingested IFN-alpha-activated regulatory splenic cell populations that may work via increased IL-4 or IL-10 production [74]. The increase of Th2-like cytokines in the NOD mouse model of T1D is in contrast to the inhibition of Th1-like cytokines in EAE and RRMS. 

Islet transplantation possesses significant potential advantages over whole-gland transplants because it is simple, may achieve insulin independence, and has clear advantages over exogenous insulin therapy. Therefore, we examined if ingested IFN-alpha, administered to islet allograft recipients, could prevent islet allograft rejection. Recipient C3H mice (H-2^k^) were made diabetic and either untreated, or treated with 10 to 1,000 IU ingested murine IFN-alpha daily from day –7 through day +14 after transplantation for a total of 21 days. Seven days after diabetes induction, recipients received allograft islets isolated from C57BL.10 donors (H-2^b^) under the kidney capsule and were followed for overt diabetes via elevated blood glucose. Control recipients and recipients fed 1,000 IU all became diabetic by day 13, whereas mice ingesting IFN-alpha had delayed rejection for up to 27 (10 IU) to 29 days (100 IU) after islet transplantation. Treatment of recipients of islet allografts with ingested IFN-alpha doubled the time period before rejection compared to control mice. The feeding period with daily IFN-alpha was doubled from 21 days to 42 days in total, seven days before transplant and 35 days after transplant. Treatment of recipients of islet allografts with prolonged ingested IFN-alpha prevented rejection in 33% of recipients 35 days post-transplant. Ingested IFN-alpha can prevent rejection if given continuously after transplantation [75].

Because there is an historical experience of a low incidence of spontaneous remission in T1D, interventions preserving beta-cell function have been used to identify positive therapeutic outcomes. We treated ten newly-diagnosed T1D patients with 30,000 IU ingested IFN-alpha within one month of diagnosis in an open-labeled phase I clinical trial and examined the difference between baseline and induced C-peptide responses respectively at 0, 3, 6, 9 and 12 months. Eight of the ten patients showed preserved beta cell function with at least a 30% increase of stimulated C-peptide levels at 0, 3, 6, 9 and 12 months after initiation of treatment. There was no discernible chemical or clinical toxicity associated with ingested IFN-alpha. There were no detectable serum increases in Th1 cytokines in these patients after IFN therapy. Ingested IFN-alpha showed potential to preserve residual betacell function in recent onset T1D [71]. 

We evaluated the safety and efficacy of ingested hrIFN-alpha for preservation of beta-cell function in young patients with recent onset T1D in a phase II randomized placebo controlled trial. Subjects ages 3–25 years diagnosed with T1D within 6 weeks of enrollment were randomized to receive ingested hrIFN-alpha at 5,000 IU, 30,000 IU, or placebo once daily for one year. The primary outcome was change in C-peptide secretion following a mixed meal. Individuals in the placebo group (n = 30) lost 56 ± 29% of their C-peptide secretion from 0 to 12 months, expressed as area under the curve (AUC) in response to a mixed meal. In contrast, children treated with hrIFN-alpha lost 29 ± 54% and 48 ± 35% (for 5000 [n = 27] and 30,000 IU [n = 31], respectively, p = 0.028, ANOVA adjusted for age, baseline C-peptide AUC and study site). Bonferroni *post-hoc* analyses for placebo *versus* 5,000 IU and placebo *versus* 30,000 IU demonstrated that the overall trend was determined by the 5,000 IU treatment group. Adverse events occurred at similar rates in all treatment groups. Ingested hrIFN-alpha was safe at the doses used. Patients in the 5,000 unit hrIFN-alpha treatment group maintained more beta-cell function one year after study enrollment compared to individuals in the placebo group, while this effect was not observed in patients who received 30,000 IU IFN-alpha. Further studies of low-dose ingested hrIFN-alpha in new-onset T1D are needed to confirm this effect.

Different cytokines are preferentially affected by ingested IFN-alpha in different disease states and species. In phase I and II studies in MS IL-2, IL-10, TGF-beta, TNF-alpha and IFN-gamma are decreased. In the NOD animal model of T1DM, IL-4 and IL-10 are increased. The different effects on cytokines in different disease states probably reflects the pleitropic effect of ingested IFN-alpha in different microenvironments [76,77]. 

Other investigators have examined oral IFN-alpha in another autoimmune disease, Sjogren's syndrome (SS). A single-blinded controlled trial was conducted to test the efficacy of low-dose oral human IFN-alpha to improve salivary function in patients with Sjogren's syndrome. Fifty-six outpatients with primary and four patients with secondary Sjogren's syndrome were assigned randomly into treatment groups of either IFN-alpha or sucralfate (non-IFN control). The IFN-alpha (150 IU) or control was given orally three times a day for six months. Saliva was quantitated monthly by the Saxon test. After six months of treatment, 15 of 30 (50%) IFN-alpha-treated patients had significantly greater saliva production increases at least 100% above baseline, compared to one of 30 (3.3%) control patients. Serial labial salivary gland biopsies of nine IFN-alpha responder patients showed that lymphocytic infiltration was significantly decreased and the proportion of intact salivary gland tissue was significantly increased after the IFN-alpha treatment [78]. Additional controlled trials in SS showed that the use of 150 IU IFN lozenges TID for 12 weeks in subjects with primary Sjogren's syndrome improved salivary output and decreased complaints of xerostomia without causing significant adverse medical events [79].

## Results

Ingested IFN-alpha shows an inverted U-shaped immunological and clinical dose-response curve (hormesis). Immune effects circumscribe the clinical effective doses in our EAE models. Inhibition of the murine *immune* system occurred at 10 and 100 IU, clinically effective doses, and also at 0.1, 1 and 1,000 IU, clinically ineffective doses. Ingested IFN-alpha significantly decreased spleen cell proliferation and IL-2 secretion at clinically ineffective low (0.1, 1 IU) and high doses (1,000 IU) [56]. However, mice fed 10 and 100 IU mIFN-alpha were protected against EAE, but animals fed 0.1, 1, and 1,000 IU were not protected despite discernable immune inhibition at these lower or higher doses [56]. Murine whole splenocytes showed upregulation of Mx mRNA after IFN-alpha ingestion of 10 and 100 IU, clinically effective doses, but not after 0, 1,000, or 5,000 IU [57]. High or very low ingested IFN-alpha doses have immune effects without a significant induction of MxA mRNA. These data show that MxA mRNA bio-induction *coincides* with EAE clinical effects, and inhibition of immune function such as proliferation and cytokine production can circumscribe doses showing clinical effects. In general, because of the U-shaped dose-response curve, there is *decreasing* (para)clinical and clinical activity with *increasing* doses of ingested hrIFN-alpha. 

## Discussion

The failure to show persistent biological effects on MRI-monitored inflammatory disease activity in our phase II MS study may be secondary to tachyphylaxis or pharmacological tolerance from an excessive non-optimal IFN-alpha dose. The results of the phase I study demonstrating immuno-modifying effects at 10,000 and 30,000, but not after 100,000 IU hrIFN-alpha2a, suggest a U-shaped dose-response curve [64]. These results and those in the Mx mRNA experiments above suggest that large quantities of ingested IFN- may not have the same effect as lesser quantities [56].

Others have noted a loss of oral type I IFN anti-viral and anti-inflammatory activity with increasing dose, demonstrating a bell shaped dose response curve. Oral administration of 1–10 IU type I IFN reduces early replication of murine cytomegalovirus (MCMV) in both the spleen and liver of MCMV-infected BALB/c mice, whereas doses from 50–1,000 were ineffective [80]. There were anti-asthmatic effects of 1–100 IU oral hIFN-beta in OVA-sensitized and challenged guinea pig asthma, but this effect was lost at 1,000 IU [81]. Lower doses of ingested IFN-alpha may paradoxically provide increased therapeutic activity.

Surprisingly, parenteral IFN-alpha also shows a U-shaped response curve. There have been repeated demonstrations of greater efficacy of IFN-alpha dose optimization using frequent scheduling. Daily therapy with parenteral IFN-alpha 10,000 IU as opposed to weekly 50,000–70,000 unit produces the most significant inhibition of human bladder, colon and pancreatic tumor growth, tumor vascularization, and matrix metalloprotease-9 mRNA after transplantation into nude mice [82,83,84].

Ascertaining optimal bio-immuno-clinico-activity of other lower dose(s) of ingested IFN-alpha2a in MS (100–3,000 IU) becomes important. There appears to be a correlation between MxA mRNA induction and clinical effects. On this basis, the dose that provides the maximum induction of MxA mRNA may potentially generate the greatest decrease of brain MRI T1 gadolinium enhancements in subsequent phase II MS clinical trials. The optimal dose of ingested IFN-alpha can be measured by examining PMNC MxA mRNA induction after ingesting 100–3,000 IU IFN-alpha2a. The dose that provides the maximal MxA induction in the most patients can be used for the phase IIb MRI/MS clinical trial.

Frequent scheduling is another option for optimizing IFN activity. In our phase II trial in T1D, we have shown proof of concept for ingested IFN-alpha. For future T1D trials and following the experience with parenteral IFN in tumor inhibition, dividing the daily 5,000 unit dose may provide more beta cell preservation. 

## Conclusions

The first phases of clinical trials using oral administration of biological agents have begun. The goals of additional trials are optimization of dosing, showing clear proof of efficacy in MS and extending the beneficial effects in T1D with frequent scheduling. 

## References

[1] Brod S.A. (1998). Hypothesis: multiple sclerosis is a type I interferon deficiency syndrome. Proc. Soc. Exp. Biol. Med..

[2] Brod S.A. (1997). Gut response. Therapy with ingested immunomodulatory proteins. Arch. Neurol..

[3] Tompkins W.A. (1999). Immunomodulation and therapeutic effects of the oral use of interferon- alpha: mechanism of action [In Process Citation]. J. Interferon Cytokine Res..

[4] Bocci V. (1990). Is interferon effective after oral administration?. J. Biol. Reg. Homeost. Agents.

[5] Wolinsky J., Appel S. (1993). Multiple Sclerosis. Current Neurology.

[6] McFarlin D., McFarland H. (1982). Multiple Sclerosis. N. Eng. J. Med..

[7] Antel J.P., Arnason B.G.W., Medof M.E. (1979). Suppressor cell function in multiple sclerosis: correlation with clinical disease activity. Ann. Neurol..

[8] Group T.I.M.S.S. (1993). Interferon beta-1b is effective in relapsing-remitting multiple sclerosis. I. Clinical results of a multicenter, randomized, double- blind, placebo-controlled trial. Neurology.

[9] Jacobs L., Cookfair D., Rudick R., Herndon R., Richert J., Salazar A., Fischer J., Granger C., Simon J., Goodkin D., Granger C., Simon J., Alam J., Bartoszak D., Bourdette D., Braiman J., Brownscheidle C., Coats M., Cohan S., Dougherty D., Kinkel R., Mass M., Munschauer F., Priore R., Pullicino P., Scherokman B., Whitham R. (1994). Intramuscular interferon beta-1a for disease progression in relapsing multiple sclerosis. The Multiple Sclerosis Collaborative Research Group (MSCRG). Ann. Neurol..

[10] Myhr K.M., Riise T., Green Lilleas F.E., Beiske T.G., Celius E.G., Edland A., Jensen D., Larsen J.P., Nilsen R., Nortvedt M.W., Smievoll A.I., Vedeler C., Nyland H.I. (1999). Interferon-alpha2a reduces MRI disease activity in relapsing-remitting multiple sclerosis. Norwegian Study Group on Interferon-alpha in Multiple Sclerosis. Neurology.

[11] Durelli L., Bongioanni M.R., Cavallo R., Ferrero B., Ferri R., Ferrio M.F., Bradac G.B., Riva A., Vai S., Geuna M. (1994). Chronic systemic high-dose recombinant interferon alfa-2a reduces exacerbation rate, MRI signs of disease activity, and lymphocyte interferon gamma production in relapsing-remitting multiple sclerosis. Neurology.

[12] Brex P.A., Gomez-Anson B., Parker G.J., Molyneux P.D., Miszkiel K.A., Barker G.J., MacManus D.G., Davie C.A., Plant G.T., Miller D.H. (1999). Proton MR spectroscopy in clinically isolated syndromes suggestive of multiple sclerosis. J. Neurol. Sci..

[13] Losseff N.A., Kingsley D.P., McDonald W.I., Miller D.H., Thompson A.J. (1996). Clinical and magnetic resonance imaging predictors of disability in primary and secondary progressive multiple sclerosis. Mult. Scler..

[14] (1995). Interferon beta-1b in the treatment of multiple sclerosis: final outcome of the randomized controlled trial. The IFNB Multiple Sclerosis Study Group and The University of British Columbia MS/MRI Analysis Group [see comments]. Neurology.

[15] (1996). Neutralizing antibodies during treatment of multiple sclerosis with interferon beta-1b: experience during the first three years. The IFNB Multiple Sclerosis Study Group and the University of British Columbia MS/MRI Analysis Group. Neurology.

[16] Brod S.A., Marshall G.D., Henninger E.M., Sriram S., Khan M., Wolinsky J.S. (1996). Interferon-beta 1b treatment decreases tumor necrosis factor-alpha and increases interleukin-6 production in multiple sclerosis. Neurology.

[17] Foulis A.K., Liddle C.N., Farquharson M.A., Richmond J.A., Weir R.S. (1986). The histopathology of the pancreas in type 1 (insulin-dependent) diabetes mellitus: a 25-year review of deaths in patients under 20 years of age in the United Kingdom. Diabetologia.

[18] Silverstein J., Maclaren N., Riley W., Spillar R., Radjenovic D., Johnson S. (1988). Immunosuppression with azathioprine and prednisone in recent-onset insulin-dependent diabetes mellitus. N. Engl. J. Med..

[19] (1993). The effect of intensive treatment of diabetes on the development and progression of long-term complications in insulin-dependent diabetes mellitus. The Diabetes Control and Complications Trial Research Group [see comments]. N. Engl. J. Med..

[20] Shah S., Malone J., Simpson N. (1989). A randomized trial of intensive insulin therapy in newly diagnosed insulin-dependent diabetes mellitus. N. Engl. J. Med..

[21] Bougneres P., Landais P., Boisson C., Carel J., Frament N., Boitard C., Chaussain J., Bach J. (1990). Limited duration of remission of insulin dependency in children with recent overt type I diabetes treated with low-dose cyclosporin. Diabetes.

[22] (1988). Cyclosporin-induced remission of IDDM after early intervention. Association of 1 yr of cyclosporin treatment with enhanced insulin secretion. The Canadian-European Randomized Control Trial Group. Diabetes.

[23] Atkison P.R., Mahon J.L., Dupre J., Stiller C.R., Jenner M.R., Paul T.L., Momah C.I. (1990). Interaction of bromocriptine and cyclosporine in insulin dependent diabetes mellitus: results from the Canadian open study. J. Autoimmun..

[24] Cook J., Hudson I., Harrison L., Dean B., Colman P., Werther G., Warne G., Court J. (1989). Double-blind controlled trial of azathioprine in children with newly diagnosed type I diabetes. Diabetes.

[25] Pozzilli P., Visalli N., Boccuni M., Baroni M., Buzzetti R., Fioriti E., Signore A., Cavallo M., Andreani D., Lucentini L. (1994). Combination of nicotinamide and steroid *versus* nicotinamide in recent-onset IDDM. The IMDIAB II Study. Diabetes Care.

[26] Lewis C., Canafax D., Sprafka J., Barbosa J. (1992). Double-blind randomized trial of nicotinamide on early-onset diabetes. Diabetes Care.

[27] Koivisto V., Aro A., Cantell K., Haataja M., Huttunen J., Karonen S., Mustajoki P., Pelkonen R., Seppala P. (1984). Remissions in newly diagnosed type 1 (insulin-dependent) diabetes: influence of interferon as an adjunct to insulin therapy. Diabetologia.

[28] www.ClinicalTrials.gov.

[29] www.ClinicalTrials.gov.

[30] Keymeulen B., Vandemeulebroucke E., Ziegler A.G., Mathieu C., Kaufman L., Hale G., Gorus F., Goldman M., Walter M., Candon S., Schandene L., Crenier L., De Block C., Seigneurin J.M., De Pauw P., Pierard D., Weets I., Rebello P., Bird P., Berrie E., Frewin M., Waldmann H., Bach J.F., Pipeleers D., Chatenoud L. (2005). Insulin needs after CD3-antibody therapy in new-onset type 1 diabetes. N. Engl. J. Med..

[31] Herold K.C., Gitelman S.E., Masharani U., Hagopian W., Bisikirska B., Donaldson D., Rother K., Diamond B., Harlan D.M., Bluestone J.A. (2005). A single course of anti-CD3 monoclonal antibody hOKT3gamma1(Ala-Ala) results in improvement in C-peptide responses and clinical parameters for at least 2 years after onset of type 1 diabetes. Diabetes.

[32] Haller M.J., Gottlieb P.A., Schatz D.A. (2007). Type 1 diabetes intervention trials 2007: where are we and where are we going?. Curr. Opin. Endocrinol. Diabetes Obes..

[33] Ludvigsson J., Faresjo M., Hjorth M., Axelsson S., Cheramy M., Pihl M., Vaarala O., Forsander G., Ivarsson S., Johansson C., Lindh A., Nilsson N.O., Aman J., Ortqvist E., Zerhouni P., Casas R. (2008). GAD treatment and insulin secretion in recent-onset type 1 diabetes. N. Engl. J. Med..

[34] Isaacs A., Lindenmann J. (1957). Virus Interference I. The interferon. Proc. R. Soc. Lond. [Biol.].

[35] Dron M., Tovey M., Baron S., Coppenhaver D.H., Dianzani F., Fleischmann W.R., Hughes T.K., Klimpel G.R., Niesel D.W., Stanton G.J., Tyring S.K. (1992). Interferon alpha/beta, gene structure and regulation. Interferon: Principles and Medical Applications.

[36] Johnson H.M., Baron S. (1977). Evaluation of the effects of interferon and interferon inducers on the immune response. Pharm. Ther..

[37] Zoon K.C. (1987). Human Interferons: Structure and Function. Interferon 9.

[38] Rashidbaigi A., Pestka S., Baron S., Dianzani F., Stanton G.J., Fleischmann W.R. (1987). Interferons: Protein Structure. Interferon System.

[39] Uze G., Lutfalla G., Knudson K.E. (1995). α and β Interferons and their receptor and their friends and relations. J. Interferon Cyt. Res..

[40] Aguet M., Mogensen K.E., Gresser I. (1983). Interferon Receptors. Interferons.

[41] Rollwagen R., Baqar S. (1996). Oral cytokine administration. Immunol. Today.

[42] Witt P.J., Goldstein D., Storer B.E., Grossberg S.E., Flashner M., Colby C.B., Borden E.C. (1992). Absense of biological effects of orally administered interferon-β_ser_. J. Interferon Res..

[43] Bocci V. (1991). Absorption of cytokines via the oropharyngeal associated lymphoid tissues - Does an unorthodox route improve the therapeutic index of interferon. Clin. Pharmacokinet.

[44] Brandtzaeg P. (1989). Overview of the mucosal immune system. Curr. Topics Microbiol. Immunol..

[45] MacDonald T.T. (1983). Immunosuppression caused by antigen feeding II. Suppressor T cells mask Peyer's patch B cell priming to orally administered antigen. Eur. J. Immunol..

[46] Mattingly J.A. (1984). Immunologic suppression after oral administration of antigen. III. Activation of suppressor-inducer cells in the Peyer's patches. Cell Immunol..

[47] Pfeffer L.M., Donner D.D. (1990). The downregulation of IFN-α receptors in human lymphoblastoid cells: relation of cellular responsiveness to antiproliferative action of IFN-α. Cancer Res..

[48] Pfeffer L.M., Colamonici O.R. (1991). Transmembrane signalling by interferon-α. Pharmac.Ther..

[49] Baron S., Coppenhaver D.H., Dianzani F., Fleischmann W.R., Hughes T.K., Klimpel G.R., Niesel D.W., Stanton G.J., Tyring S.K., Baron S., Coppenhaver D.H., Dianzani F., Fleischmann W.R., Hughes T.K., Klimpel G.R., Niesel D.W., Stanton G.J., Tyring S.K. (1992). Introduction to the interferon system. Interferon: Principles and Medical Applications.

[50] Fleischmann W.R., Koren S., Fleischmann C.M. (1992). Orally administered interferons exert their white blood cell suppressive effects via a novel mechanism. Proc. Soc. Exp. Biol. Med..

[51] Cantell K., Pyhala L. (1973). Circulating interferon in rabbits after administration of human interferon by different routes. J. Gen. Virol..

[52] Gibson D.M., Cotler S., Spiegel H.E., Colburn W.A. (1985). Pharmacokinetics of recombinant leukocyte A interferon following various routes and modes of administration to the dog. J. Interferon Res..

[53] Wills R.J., Spiegel H.E., Soike K.F. (1984). Pharmacokinetics of recombinant alpha A interferon following I.V. infusion and bolus, I.M., and P.O. administrations to African green monkeys. J. Interferon Res..

[54] Horisberger M.A., Baron S., Coppenhaver D.H., Dianzani F., Fleischmann W.R., Hughes T.K., Klimpel G.R., Niesel D.W., Stanton G.J., Tyring S.K. (1992). Mx protein: Function and mechanism of action. Interferon: Principles and Medical Applications.

[55] Roers A., Hochkeppel H., Horisberger M., Hovanessian A., Haller O. (1994). MxA gene expression after live virus vaccination: A sensitive marker for endogenous type I interferon. J. Infect. Dis..

[56] Brod S.A., Khan M. (1996). Oral administration of IFN-alpha is superior to subcutaneous administration of IFN-alpha in the suppression of chronic relapsing experimental autoimmune encephalomyelitis. J. Autoimmun..

[57] Brod S.A., Nelson L., Jin R., Wolinsky J.S. (1999). Ingested interferon alpha induces Mx mRNA. Cytokine.

[58] Brod S.A., Khan M., Bright J., Sriram S., Marshall G.D., Henninger E.M., Kerman R.H., Wolinsky J.S. (1995). Decreased CD3-mediated interferon-gamma production in relapsing- remitting multiple sclerosis. Ann. Neurol..

[59] Brod S.A., Khan M., Nelson L.D., Decuir B., Malone M., Henninger E. (2000). Adoptive transfer from interferon-alpha-fed mice is associated with inhibition of active experimental autoimmune encephalomyelitis by decreasing recipient tumor necrosis factor-alpha secretion. J. Immunother..

[60] Brod S.A., Scott M., Burns D.K., Phillips J.T. (1995). Modification of acute experimental autoimmune encephalomyelitis in the Lewis rat by oral administration of type 1 interferons. J. Interferon Cytokine Res..

[61] Brassard D.L., Grace M.J., Bordens R.W. (2002). Interferon-alpha as an immunotherapeutic protein. J. Leukoc. Biol..

[62] Xing T., Zhang L., Lu Q., Hou J., Feng X., Luo K. (2001). Th1/Th2 type cytokines in hepatitis B patients treated with interferon-alpha. Chin. Med. J. (Engl.).

[63] Monteleone G., Pender S.L., Wathen N.C., MacDonald T.T. (2001). Interferon-alpha drives T cell-mediated immunopathology in the intestine. Eur. J. Immunol..

[64] Brod S.A., Kerman R.H., Nelson L.D., Marshall G.D., Henninger E.M., Khan M., Jin R., Wolinsky J.S. (1997). Ingested IFN-alpha has biological effects in humans with relapsing- remitting multiple sclerosis. Mult. Scler..

[65] Isaac C., Li K.B., Genton M., Jardine C., Grochowski E., Palmer M., Kastruloff L.F., Oger J.J., Paty D.W. (1988). MS: A serial study using MRI in relapsing patients. Neurology.

[66] Miller D.H., Barkhof F., Berry I., Kappos L., Scotti G., Thompson A.J. (1991). MR imaging in monitoring the treatment of multiple sclerosis: Concerted action guidelines. J. Neurol. Neurosurg. Psychiat..

[67] Paty D.W., Oger J.J., Kastruloff L.F., Hashimoto S.A., Hooge J.J., Eisen A.A., Eisen K.A., Purves S.J., Low M., Brangjes V., Robertson W., Li D.B.K. (1989). Biologic *vs.* clinical MS. Neurology.

[68] Thompson A.J., Kermode A.J., MacManus D.G., Kendall B.E., Kingsley D.P.E., Moseley I.F., McDonald W.I. (1990). Patterns of disease activity in MS: clinical and MRI study. Br. Med. J..

[69] Thompson A.J., Miller D., Youl B., MacManus D., Moore S., Kingsley D.P.E., Kendall B.E., Feinstein A., McDonald W.I. (1993). Serial gadolinium enhanced MRI in RR MS of varying didease duration. Neurology.

[70] Willoughby E.W., Grochowski E., Li D.K., Oger J., Kastrukoff L.F., Paty D.W. (1989). Serial magnetic resonance scanning in multiple sclerosis: a second prospective study in relapsing patients. Ann. Neurol..

[71] Brod S., Orlander P., Lavis V., Brosnan P., Hardin D., Henninger E., Nyugen M., Riley W. (2001). Ingested IFN-α prolongs the “honeymoon” period in newly diagnosed type I diabetes mellitus. J. Interferon Cytokine Res..

[72] Calabrese E.J. (2005). Hormetic dose-response relationships in immunology: occurrence, quantitative features of the dose response, mechanistic foundations, and clinical implications. Crit. Rev. Toxicol..

[73] Brod S.A., Nguyen M., Hood Z., Shipley G.L. (2006). Ingested (oral) IFN-alpha represses TNF-alpha mRNA in relapsing-remitting multiple sclerosis. J. Interferon Cytokine Res..

[74] Brod S., Darcan S., Malone M., Pappolla M., Nelson L. (1998). Ingested IFN-α suppresses IDDM in the NOD mouse. Diabetologia.

[75] Brod S.A., Katz S., Phan T., Stepkowski S. (2000). Ingested interferon-alpha prevents allograft islet transplant rejection. Transplantation.

[76] Daynes R.A., Araneo B.A., Dowell T.A., Huang K., Dudley D. (1990). Regulation of murine lymphokine production *in vivo*. III. The lymphoid tissue microenvironment exerts regulatory influences over T helper cell function. J. Exp. Med..

[77] Bocci V. (1988). Roles of interferon produced in physiological conditions. A speculative review. Immunology.

[78] Shiozawa S., Tanaka Y., Shiozawa K. (1998). Single-blinded controlled trial of low-dose oral IFN-alpha for the treatment of xerostomia in patients with Sjogren's syndrome. J. Interferon Cytokine Res..

[79] Ship J.A., Fox P.C., Michalek J.E., Cummins M.J., Richards A.B. (1999). Treatment of primary Sjogren's syndrome with low-dose natural human interferon-alpha administered by the oral mucosal route: a phase II clinical trial. IFN Protocol Study Group. J. Interferon Cytokine Res..

[80] Bosio E., Beilharz M.W., Watson M.W., Lawson C.M. (1999). Efficacy of low-dose oral use of type I interferon in cytomegalovirus infections *in vivo.*. J. Interferon Cytokine Res..

[81] Satoh Y., Kasama K., Kuwabara M., Yimin, Diao H.Y., Nakajima H., Kohanawa M., Minagawa T. (1999). Suppression of late asthmatic response by low-dose oral administration of interferon-beta in the guinea pig model of asthma. J. Interferon Cytokine Res..

[82] Slaton J.W., Perrotte P., Inoue K., Dinney C.P., Fidler I.J. (1999). Interferon-alpha-mediated down-regulation of angiogenesis-related genes and therapy of bladder cancer are dependent on optimization of biological dose and schedule. Clin. Cancer Res..

[83] Ozawa S., Shinohara H., Kanayama H.O., Bruns C.J., Bucana C.D., Ellis L.M., Davis D.W., Fidler I.J. (2001). Suppression of angiogenesis and therapy of human colon cancer liver metastasis by systemic administration of interferon-alpha. Neoplasia.

[84] Solorzano C.C., Hwang R., Baker C.H., Bucana C.D., Pisters P.W., Evans D.B., Killion J.J., Fidler I.J. (2003). Administration of optimal biological dose and schedule of interferon alpha combined with gemcitabine induces apoptosis in tumor-associated endothelial cells and reduces growth of human pancreatic carcinoma implanted orthotopically in nude mice. Clin. Cancer Res..

